# Exercise improves bone formation by upregulating the Wnt3a/β-catenin signalling pathway in type 2 diabetic mice

**DOI:** 10.1186/s13098-021-00732-6

**Published:** 2021-10-23

**Authors:** Xianghe Chen, Kang Yang, Peng Sun, Renqing Zhao, Bo Liu, Pengcheng Lu

**Affiliations:** 1 College of Physical Education, Yangzhou University, Yangzhou, 225127 Jiangsu China; 2 Rehabilitation Medicine Department, Northern Jiangsu People’s Hospital Affiliated to Yangzhou University, Yangzhou, 225001 Jiangsu China; 3 College of Physical Education and Health, East China Normal University, Shanghai, China

**Keywords:** Exercise, Bone formation, Wnt3a, β-catenin, Type 2 diabetes, Osteoblast

## Abstract

**Background:**

The bone formation ability of type 2 diabetes is inhibited, and exercise can effectively improve the bone formation of T2DM. However, whether exercise can mediate the Wnt3a/β-catenin pathway to improve the mechanism of bone formation and metabolism still needs further research.

**Methods:**

A T2DM mouse model was established by a high-fat diet and STZ injection, and the mice were trained with swimming and downhill running exercise. Alizarin red staining is used to observe the changes of the left femoral trabecular bone; micro-CT is used to analyze the trabecular and cortical BMD, BV/TV, BS/BV, BS/TV, Tb.Th, Tb.Sp; the ALP staining of skull was used to observe the changes in ALP activity of bone tissues at the skull herringbone sutures; ALP staining was performed to observe the changes in the number of OBs and ALP activity produced by differentiation; Quantitative PCR was used to detect mRNA expression; Western blot was used to detect protein expression levels.

**Results:**

When the Wnt3a/β-catenin pathway in the bones of T2DM mice was inhibited, the bone formation ability of the mice was significantly reduced, resulting in the degradation of the bone tissue morphology and structure. Swimming caused the significant increase in body weight and Runx2 mRNA expression, while downhill running could significantly decrease the body weight of the mice, while the tibia length, wet weight, and the trabecular morphological structure of the distal femur and the indexes of bone histomorphology were significantly improved by activating the Wnt3a/β-catenin pathway.

**Conclusions:**

Bone formation is inhibited in T2DM mice, leading to osteoporosis. Downhill running activates the Wnt3a/β-catenin pathway in the bones of T2DM mice, promotes OB differentiation and osteogenic capacity, enhances bone formation metabolism, and improves the bone morphological structure.

## Introduction

Type 2 diabetes mellitus (T2DM) is an energy metabolic disorder characterised by high blood glucose and reduced insulin secretion, a pathogenesis closely related to insulin resistance and impaired islet β cell function [17]. Metabolic disorders in type 2 diabetes lead to many complications, such as microangiopathy, diabetic neuropathy, and others. [18]. As observed by Deborah et al. patients with T2DM had a lower bone quality and a higher risk of bone fracture [37]. Additionally, the bone tissue microstructure, such as the trabecular number (Tb.N), biomechanical properties, bone mineral density (BMD) and bone volume fraction (BV/TV) were found to be significantly lower in T2DM mice than in normal mice [22, 39]. Huang et al. [22] reported that the BMD, BV/TV, and Tb.N of the femur were significantly decreased in T2DM mice. The aforementioned results revealed that T2DM caused a decrease in bone mass and the degeneration of bone tissue morphology.

Osteoblasts (OBs) are differentiated from bone marrow mesenchymal stem cells (BMSCs), and there is a close positive correlation between the number of OBs, osteogenic capacity and bone formation metabolism [34]. However, there is a scarcity of related studies on how T2DM inhibits OB differentiation and bone formation. Park et al. [35] verified that the number of OBs differentiated by BMSCs in T2DM mice and the bone formation thereof decreased, and many signalling pathways or key molecules have a significant regulatory function in the process. Wnt3a/β-catenin is a key pathway regulating bone formation, and can regulate OB differentiation and osteogenic capacity [13, 21]. As a key subtype of Wnt proteins, activated Wnt3a leads to the phosphorylation of β-catenin, which activates the expression of downstream target genes Runx2, Osx, and others, while regulating OB differentiation and bone formation [44]. However, whether the Wnt3a/β-catenin pathway has a regulatory function in T2DM inhibition of OB differentiation and bone formation has not yet been revealed.

Exercise is an important method to improve bone formation, which can promote OB differentiation and osteogenic capacity, and improve bone formation metabolism [15, 23]. Despite such benefits, the mechanical stimulation of the bone by different modes of exercise (divided into direct force, that is, the reaction of the ground to the bone and indirect force, that is, the pulling force of the muscle on the bone) are considerably different [20]. In a related study, findings were made that the effect of direct forces on bone formation was significantly better than indirect forces [2]. Although there are many existing studies based on the improvement of bone metabolism in T2DM by exercise, and several studies have focused on bone phenotypic indicators such as BMD and bone biomechanics [5, 19], no related studies on the effects of different exercises on the expression of Wnt3a/β-catenin signalling pathway-related molecules and bone formation in T2DM mice have been conducted.

In the present study, a high-fat-diet (HFD) combined with a one-time injection of streptozotocin was adopted, so as to establish a T2DM mouse model to evaluate the effects of different exercises on OB differentiation, osteogenic capacity and bone tissue microstructure. The results reveal that downhill running activated the Wnt3a/β-catenin pathway in the bones of T2DM mice to promote OB differentiation and bone formation capacity, promote bone formation metabolism in type 2 diabetic mice, and improve the microstructure of the bone tissue, with the effect being better than swimming.

## Materials and methods

### Animals

Forty four-week-old C57BL/6 mice were provided by B&K Laboratory Animal Company (Shanghai, China). The mice were housed under standard conditions and the room was maintained on a cycle of 12 h light and 12 h darkness, at a temperature of 25  ±  3 °C and with free access to food and water. All animal experiments were approved by the Ethics Committee on Animal Use of Yangzhou University.

### Establishment of the T2DM model

All animals were adaptively fed for 1 week and then randomly divided into a normal group (ZC, n  =  10) and a T2DM model (n  =  30). The T2DM model mice were fed a high-fat diet (31.7% lard, 25.8% casein 30 mesh, 16.3% maltodextrin 10, 9% sucrose, 6.5% cellulose BW200, 3.4% soybean oil, 2.3% potassium citrate, 1% H_2_O, 1.6% mineral mix S10026, 1.4% dicalcium phosphate, 1.3% vitamin mix V10001, 0.8% calcium carbonate, 0.4% L-cystine, and 0.3% choline bitartrate, purchased from Jiangsu Xietong Pharmaceutical Bio-engineering Co., Ltd.) for 6 weeks, and then STZ (80 mg/kg) was injected 12 h after fasting. The normal group mice were injected with a citric acid-sodium citrate solution. The blood glucose concentration of the mice was detected after 12 h of fasting 2 weeks later. The blood glucose concentration was above 8 mmol/L for the T2DM mice [24, 46], and 27 models were successfully established and randomly divided into the T2DM control group (TC, n  =  9), the T2DM swimming group (TS, n  =  9) and the T2DM downhill running group (TD, n  =  9). Normal mice were fed a normal diet, and T2DM mice continued to have a high-fat diet, with both groups drinking water freely.

### Training protocol

TS group and TD group mice were subjected to swimming and downhill running, respectively. For the swimming group, the mice were placed in a (42 cm long × 40 cm wide × 36 cm water depth) container, for 50 min/day, 8 weeks in total. The first week was adaptive training, 30 min a day for the first 2 days, 40 min a day for the 3rd and 4th days, then 50 min a day for the 5th and 6th days, while normal training started in the 2nd week. The conditions for downhill running included: 0.8 km/h, 50 min, slope  − 9 degrees, 6 days/week, a total of 8 weeks. The first week was adaptive training, 30 min a day for the first 2 days, 40 min a day for the 3rd and 4th days, then 50 min a day for the 5th and 6th days. Later, normal training started in the 2nd week.

### Body weight

The body weights of each group of mice were weighed using an electronic scale.

### Oral glucose tolerance test (OGTT)

After the last exercise intervention in the eighth week, the mice were fasted for 12 h and gavaged 2 g/kg glucose for the OGTT experiment. After gavage at 0, 30, 60, and 120 min, blood glucose was measured through the tail vein, and the area under the curve was calculated. The calculation method of the area under the time of the blood glucose curve was AUC (h mmol L^−1^) = 1/4 A + 1/2 B + 1/2 C + 1/4 D (A, B, C, and D were the blood glucose values measured after 0, 30, 60 and 120 min of intragastric glucose administration) [28].

### Measurement of bone length and wet weight

A Vernier caliper was used to measure the left tibia lengths of the mice in each group. An electronic balance was used to measure the left tibia wet weights of the mice in each group.

### Alizarin red staining

After fixing with 4% PFA for 24 h, the left femurs were decalcified in 4% EDTA for 30 days after washing with PBS. Subsequently, alcohol gradient dehydration was conducted and the left femurs were embedded in paraffin, before being sagittally cut into 6 μm sections. After placing on a baking sheet, the slices were dewaxed with xylene, and rehydrated with an alcohol gradient (from a high to a low concentration). The slices were washed in PBS solution and Alizarin red staining was performed for 5 min followed by washing with PBS. Then, dehydration was performedwith an alcohol gradient (a low to a high concentration), xylene I and II for 5 min each, before sealing with neutral gum. Finally, a Leica microscope was used to take photographs.

### Microcomputed tomography (micro-CT) analysis

The left femur of each group was fixed in 4% paraformaldehyde (PFA) for 24 h, washed with PBS and then scanned by a micro-CT scanner (Skyscan, Aarselaar, Belgium) with a resolution of 18 μm per pixel. For 3-dimensional histomorphometric analysis of the trabecular and cortical bone, cross-sectional images of the distal femur were used. The region of interest (ROI) of the distal femur selected for analysis was 5% of the femoral length from 0.05 mm below the growth plate to determine the trabecular and cortical BMD, BV/TV, bone surface area to volume ratio (BS/BV), ratio of bone surface area to tissue volume (BS/TV), trabecular thickness (Tb.Th), Tb.N and trabecular separation (Tb.Sp).

### ALP staining of the skull

After washing with PBS, the skull was fixed in 4% PFA at 4 degrees for 24 h. Then, 0.1% Triton-X100 (for permeabilisation) and PBST were used to treat the skull in a 4 degree environment for 24 h. The skull was stained with an ALP dye solution (0.002 g AS-MAX and 0.006 g Fast Red Violet LB Salt dissolved in 5 ml pH 8.3 of Tris–HCl and 5 ml of ddH2O) for 1 h. A camera (Canon, Japan) was used to take photographs and Photoshop software was used to take a screenshot of the seam locations.

### ALP and Alizarin red staining of OBs

After euthanising the mice under aseptic conditions, the bilateral femurs and tibias were taken from each mouse and cleaned of all attached soft tissues. The bone marrow cavity was exposed, and the bone marrow was isolated. A single-cell suspension was prepared by repeated aspiration. The total bone marrow cells were counted by haemocytometer and cultured in alpha minimal essential medium (α-MEM; GIBCO, USA) supplemented with 10% foetal calf serum (GIBCO, USA), 1000 U/ml ciprofloxacin (Sigma, USA). The cells were disseminated into 6-well plates (Costar, USA) at a concentration of 1 × 107 cells/well in 2 mL α-MEM, and incubated at 37 °C in a humidified atmosphere of 5% CO_2_ in air. The medium was changed every other day to remove the non-adherent cells. On the 7th day of culture, 100 U/ml glycerophosphoric acid (Sigma) and 1000 U/ml ascorbic acid (Sigma) were added to the α-MEM to induce the bone marrow cells to differentiate into osteoblasts. The medium was changed every other day. On the 7th day after differentiation, the alkaline phosphatase positive colony forming units-fibroblastic (ALP + CFU-f) were fixed with 10% PFA for 10 min at RT. After washing with PBS, the cells were stained with ALP and Alizarin red dye solution. A camera (Canon, Japan) was used to photograph the staining results.

### Quantitative PCR

A portion of the right femur (with the bone marrow removed) of the same weight from each mouse was placed in a grinding tube, and pre-soaked steel beads in DEPC water and TRIzol (Takara, Shiga, Japan) were added. Tissue grinding was performed to extract the total RNA. Subsequently, using a reverse transcriptase cDNA kit (Takara, Shiga, Japan), the total RNA was reverse transcribed into cDNA. The mRNA expression of related factors was evaluated with a Real-Time PCR System (Applied Biosystems 7500, Waltham, MA, USA) by using a SYBR Premix Ex Taq kit (Takara, Shiga, Japan). The primers were synthesised by Sangon Biotech Co., Ltd. (Shanghai, China) (Wnt3a, Forward: 5′-AGGACCCATCTGATTCCCCA-3′ and Reverse: 5′-CTTGTGGCAGATGGGCTG TA-3′, β-catenin, Forward: 5′-AGACAGCTCGTTGTACTGCT-3′ and Reverse: 5′ GTGTCGTGATGGCGTAGAAC-3′, Runx2, Forward: 5′-GTCCTATGACCAGTCTTA CC-3′ and Reverse: 5′-GATGAAATGCCTGGGAACTG-3′, Osx, Forward: 5′-GTTCACCTGTCTGCTCTGCTC-3′ and Reverse: 5′-AGCTCCTTAGGG CCACTTGG-3′, β-actin: Forward: 5′-ACCCAGAAGACTGTGGATGG-3′ and Reverse: 5′- TTCAGCTCAGGGATGACCTT-3′). Each gene was analysed in three repetitions. The method of 2^−ΔΔCt^ was used to calculate the mRNA expression of each gene.

### Western blot

Extraction of total bone tissue protein and the procedures and methods for detecting the cytokines protein expression in bone were performed in accordance with a previous study [29]. The antibodies involved in the experiments were directed against Wnt3a (CST, USA, RRID:AB_2215411), β-catenin (CST, USA, RRID:AB_11127855), Runx2 (CST, USA, RRID:AB_10949892) and Osx (abcam, USA, AB_22552), which were derived from rabbits. The dilution ratio of all of the antibodies was 1:1000. Blots were tested by the Alpha gel imaging system (AlphaImager HP, USA) and Quantity One software (Bio-Rad Inc., USA) was used to determine the grey values. The ratio of the grey values of target proteins to the internal reference is defined as the expression of the target proteins.

### Statistical analysis

The experimental results are presented as the mean  ±  standard deviation (SD). The data were statistically analysed by SPSS 20.0 (The ZC group and the TC group were subjected to independent sample t tests. One-way analysis of variance was used for the TC, TS and TD groups), P < 0.05 and P < 0.01, respectively, indicated that the experimental results had significant differences.

## Results

### Effect of exercise on OGTT in T2DM mice

**Fig. 1:**
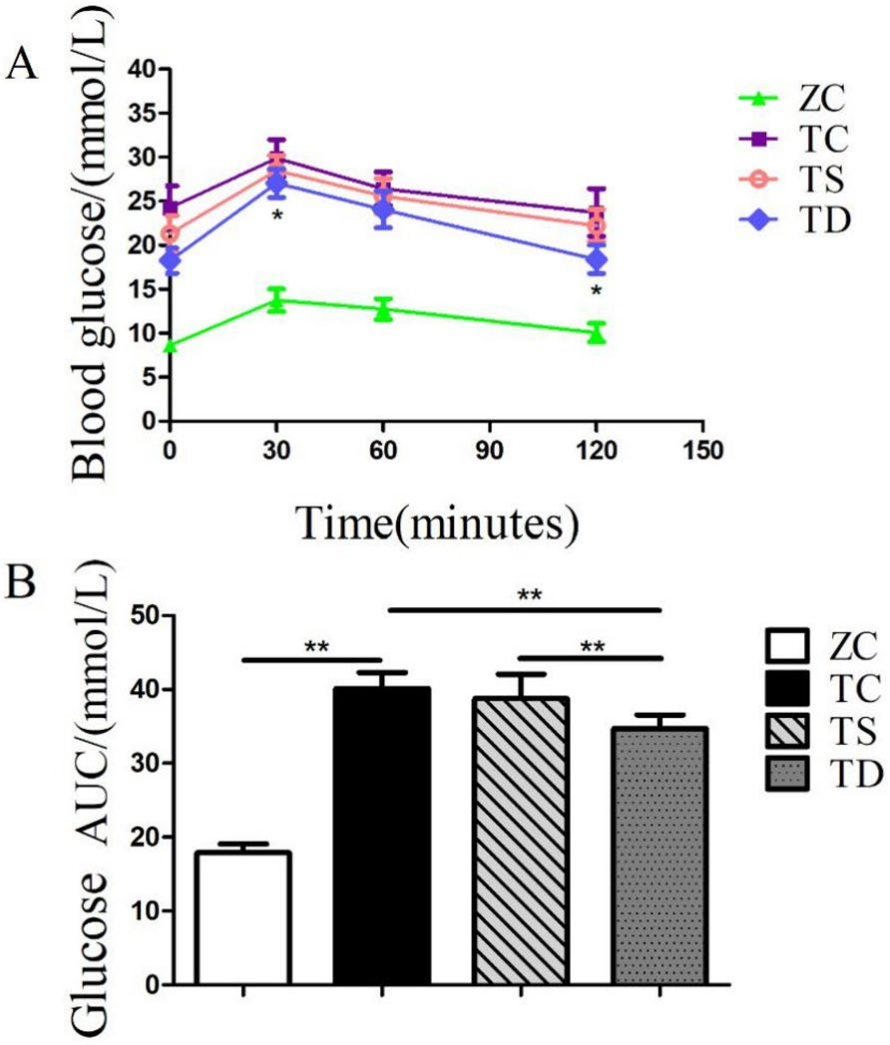
The effect of exercise on oral glucose tolerance test (OGTT) in diabetic mice. The data in the graphs are expressed as the mean  ±  SD, n  =  6. **A** Oral glucose tolerance curve in the ZC (n  =  10), TC (n  =  9), TS (n  =  9), TD (n  =  9) groups. **B** Area under the glucose curve in **A**. *P  <  0.05, **P  <  0.01

Oral glucose tolerance tests (OGTT) reflect the ability of the body to regulate blood sugar [6]. An observation can be made from Fig. 1A that the blood glucose concentration of mice in the ZC group reached a maximum of 13.7 mmol/L after 30 min of intragastric administration, and within 120 min, the blood glucose concentration was restored to the basic level of 10.06 mmol/L. The blood glucose concentration of the mice in the TC group increased to a maximum of 29.87 mmol/L within 30 min, and there was no significant drop to 23.72 mmol/L at 120 min, indicating that the islet function and the ability to regulate blood glucose of the mice in the TC group were impaired (Fig. 1A). Glucose tolerance was severely impaired. The glucose tolerance of the TS group did not change significantly compared with the TC group. After intragastric glucose, the blood glucose concentration increased to a maximum of 28.47 mmol/L within 30 min. However, there was no significant decrease to 22.23 mmol/L at 120 min (Fig. 1A). Notably, compared with the TC group, the glucose tolerance in the TD group was significantly increased (P < 0.01), which was consistent with the results of AUC analysis. The AUC in the TD group was significantly decreased (P < 0.01) compared with the TS group, indicating that downhill running could reduce the rate of glucose absorption and improve the impaired glucose tolerance of diabetic mice (Fig. 1B).

### Exercise decreased the body weight, bone length and bone wet weight of the T2DM mice

**Fig. 2:**
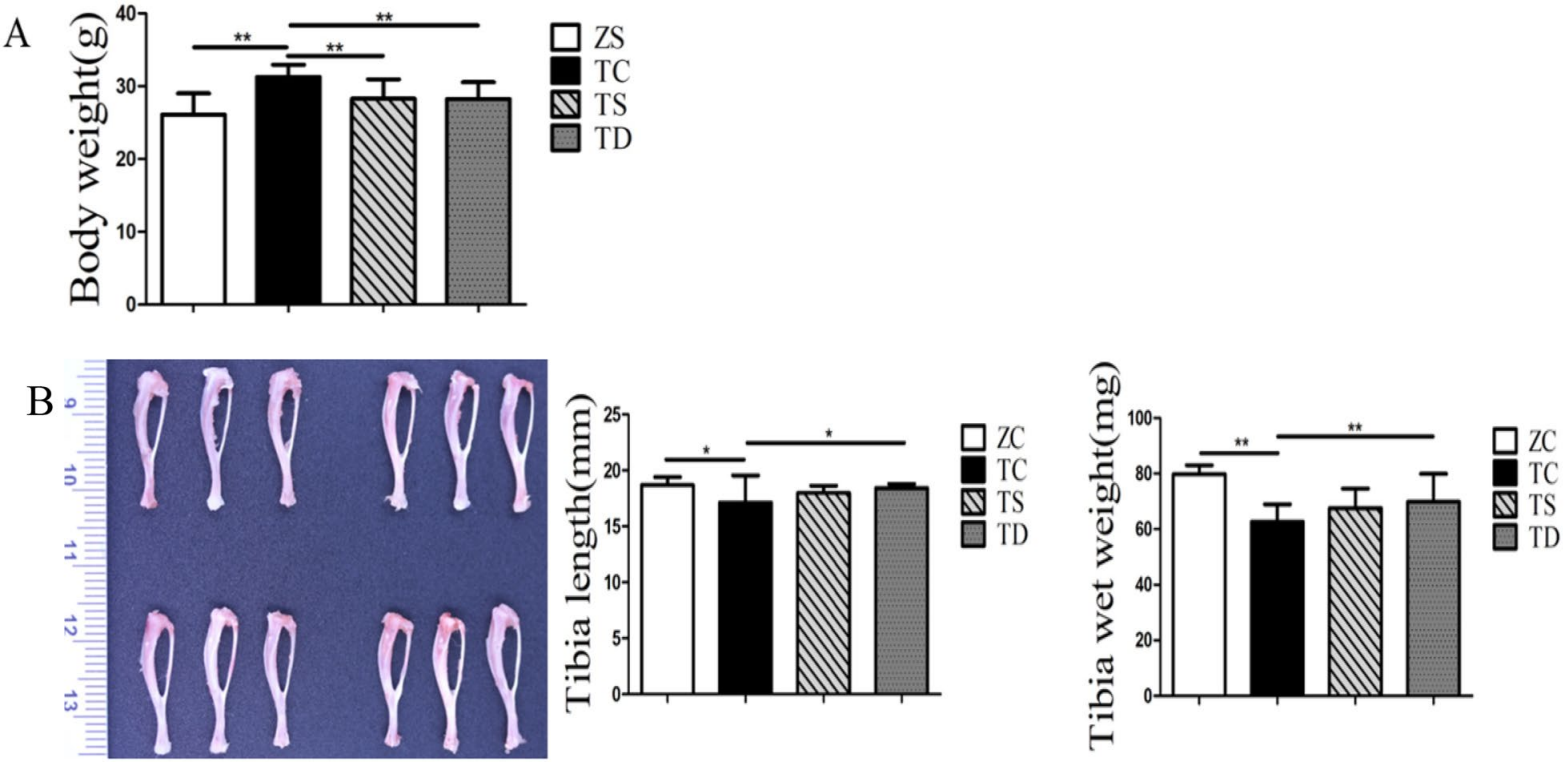
Effects of exercise on body weight and tibia length and wet weight. The data in the graphs are expressed as the mean  ±  SD, n  =  6. **A** The effects of T2DM on the body weight of mice and the effects of swimming and downhill running on the body weight of T2DM mice. **B** The effects of T2DM on tibia length and wet tibia weight in mice. Swimming and downhill running interventions for 8 weeks, tibia length and tibia wet weight changes in T2DM mice. *P  <  0.05, **P  <  0.01

To further investigate the changes in body weight, bone length and wet weight of the T2DM mice, electronic scales, electronic balances and Vernier callipers were used to measure said indexes. The body weight of the TC group was significantly increased relative to the ZC group (P < 0.01). Comparedwith the TC group, the body weight of the TS group was obviously lower (P < 0.01), while that of the TD group was further reduced (P < 0.01) (Fig. 2A). The bone length of the TC group was less than that of the ZC group (P < 0.05), indicating that downhill running could significantly promote the tibia to grow longer (P < 0.05), but the effect of swimming on tibia length was not obvious (P > 0.05) (Fig. 2B). The wet weight of the tibia in the TC group was significantly lower than in the ZC group (P < 0.01). Compared with the TC group, the tibia wet weight of the TS group had no significant difference, but the TD group was significantly increased (Fig. 2B).

The aforementioned results reveal that T2DM could promote the body weight and affect the bone length and wet weight. Exercise could significantly reduce the body weight of T2DM mice, and downhill running had a better effect on bone length and wet weight than swimming.

### Exercise improved the bone histomorphology of the T2DM mice

**Fig. 3:**
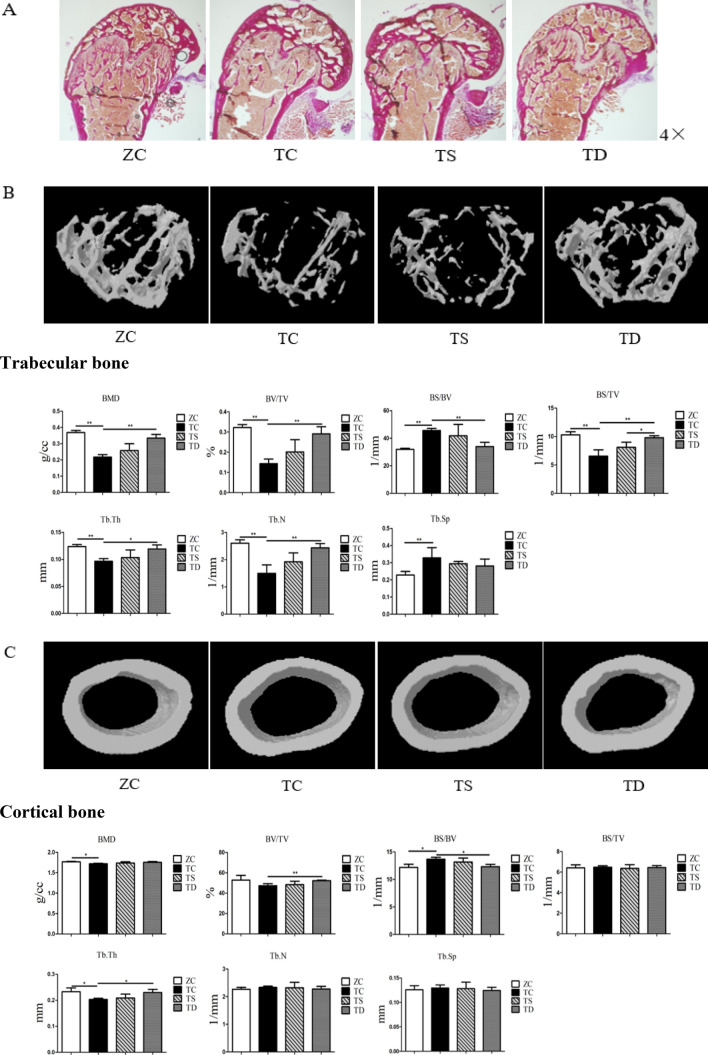
Effects of exercise on the morphological structure of bone. The data in the graphs are expressed as the mean  ±  SD, n  =  6. **A** Using Alizarin red staining to observe the effects of two kinds of exercise on the changes of trabecular bone in the distal femoral cancellous bone of T2DM mice (4 ×). **B** Representative images of micro-CT analysis of the microstructure of the trabecular bone at the mouse distal femur and related indicator changes. *BMD* bone mineral density; *BV/TV* bone volume fraction; *BS/BV* bone surface area to bone volume ratio; *BS/TV* ratio of bone surface area to tissue volume; *Tb.Th* trabecular thickness; *Tb.N* trabecular number; *Tb.Sp* trabecular separation. **C** Representative images of micro-CT analysis of the microstructure of the cortical bone at the mice distal femur and related indicator changes. *P  <  0.05, **P  <  0.01

Bone histomorphology is a significant index in the evaluation of bone metabolism, which is impaired by T2DM [14]. To evaluate the effect of exercise on bone histomorphology, Alizarin red staining and micro-CT of the distal femur were performed. Compared with the ZC group, the trabecular bone histomorphology of the TC group was significantly degraded. Further, the BMD (P < 0.05), BS/BV (P < 0.05), Tb.Th (P < 0.05) of the cortical bone and BMD (P < 0.01), BV/TV (P < 0.01), BS/BV (P < 0.01), BS/TV (P < 0.01), Tb.Th (P < 0.01), Tb.N (P < 0.01) and Tb.Sp (P < 0.01) of the trabecular bone of the TC group were significantly changed, while the BV/TV (P > 0.05), BS/TV (P > 0.05), Tb.N (P > 0.05), and Tb.Sp (P > 0.05) of the cortical bone exhibited no significant difference (Fig. 3). The aforementioned results reveal that the morphology of cancellous bone tissue in T2DM mice was significantly degraded, but the cortical bone changes were not significant.

Exercise had a significant impact on bone histomorphology, and exercise could increase BMD and improve the related indicators of bone tissue microstructure [11, 43]. In the present study, there were no significant differences between the TC and TY group. The trabecular bone histomorphology of the TD group was notably improved. Compared with the TC group, the BMD (P > 0.05), BV/TV (P > 0.05), BS/BV (P > 0.05), BS/TV (P > 0.05), Tb.Th (P > 0.05), Tb.N (P > 0.05), and Tb.Sp (P > 0.05) of the trabecular and cortical bone in the TY group exhibited no significant changes, while the BV/TV (P < 0.01), BS/BV (P < 0.05), and Tb.Th (P < 0.05) of the cortical bone and BMD (P < 0.01), BV/TV (P < 0.01), BS/BV (P < 0.01), BS/TV (P < 0.01), Tb.Th (P < 0.05), and Tb.N (P < 0.01) of the trabecular bone in the TD group were all significantly changed; however, the BMD (P > 0.05), BS/BV (P > 0.05), Tb.N (P > 0.05), and Tb.Sp (P > 0.05) of the cortical bone and Tb.Sp (P > 0.05) of the trabecular bone were not changed (Fig. 3). The BS/TV (P < 0.05) of the trabecular bone in the TD group was significantly improved relative to the TY group, and other relevant indicatorsexhibited no significant differences. The aforementioned results indicate that downhill running could improve the bone histomorphology of T2DM mice significantly, but the effect of swimming was not obvious.

### Exercise significantly promoted OBs and the osteogenic capacity of T2DM mice

**Fig. 4:**
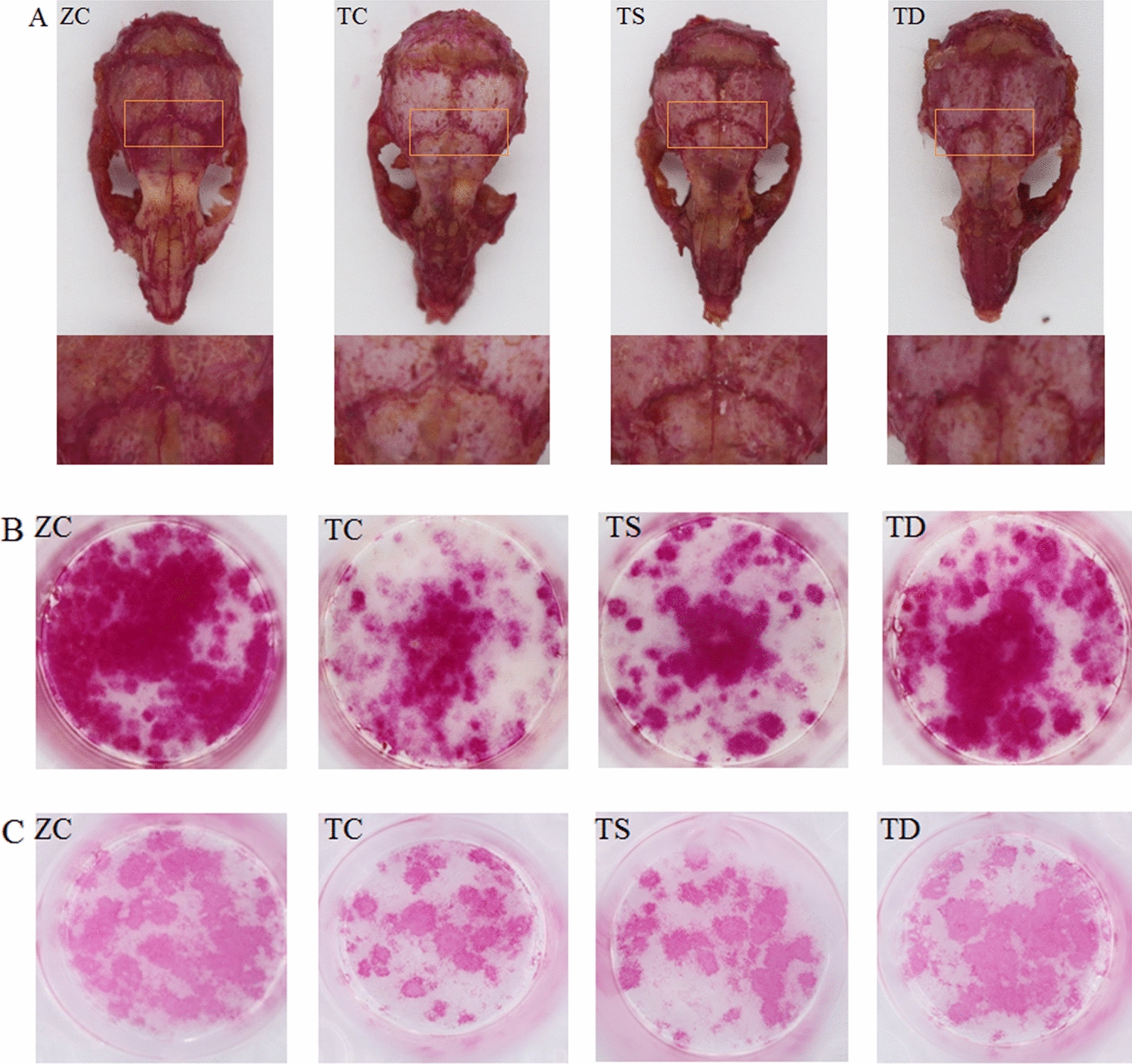
Effects of exercise on bone formation capacity. **A** ALP staining was performed on the skulls of mice in each group to observe the changes in ALP activity of bone tissues at the skull herringbone sutures, with a darker colour indicating stronger ALP activity. **B** After inducing BMSCs to differentiate into OBs, ALP staining was performed to observe the changes in the number of OBs and ALP activity produced by differentiation. **C** Alizarin red staining of the OBs produced by induced differentiation was performed to observe the changes in the number of OBs produced by differentiation and bone formation ability

OBs are differentiated from BMSCs, and the osteogenic capacity of OBs will directly determine bone metabolism [10]. Type 2 diabetes is an energy metabolic disease that causes a significant decrease in OB differentiation and osteogenic capacity [33]. In the present study, as revealed by skull and OB Alizarin red staining and OB ALP staining, the number of OBs and the osteogenic capacity of type 2 diabetic mice were significantly decreased. Exercise is a significant factor in promoting OB differentiation and improving osteogenic capacity [31]. However, there is a scarcity of studies on the effects of exercise on OB differentiation and osteogenic capacity in type 2 diabetic mice. After 8 weeks of training, the changes in the ALP staining of the skull and OBs and the Alizarin red staining of OBs in the TS group were not significant, but the related results of the TD group were significantly improved (Fig. 4). In addition, there were significant differences between the TS group and the TD group. The aforementioned research results reveal nthat T2DM resulted in a significant decrease in the number of OBs and osteogenic capacity. Exercise could promote BMSCs to differentiate into OBs and improve the osteogenic capacity, and the effect of downhill running was better than swimming (Fig. 4).

### Exercise caused a significant increase of the related factors in the Wnt3a/β-catenin pathway of T2DM mice

**Fig. 5:**
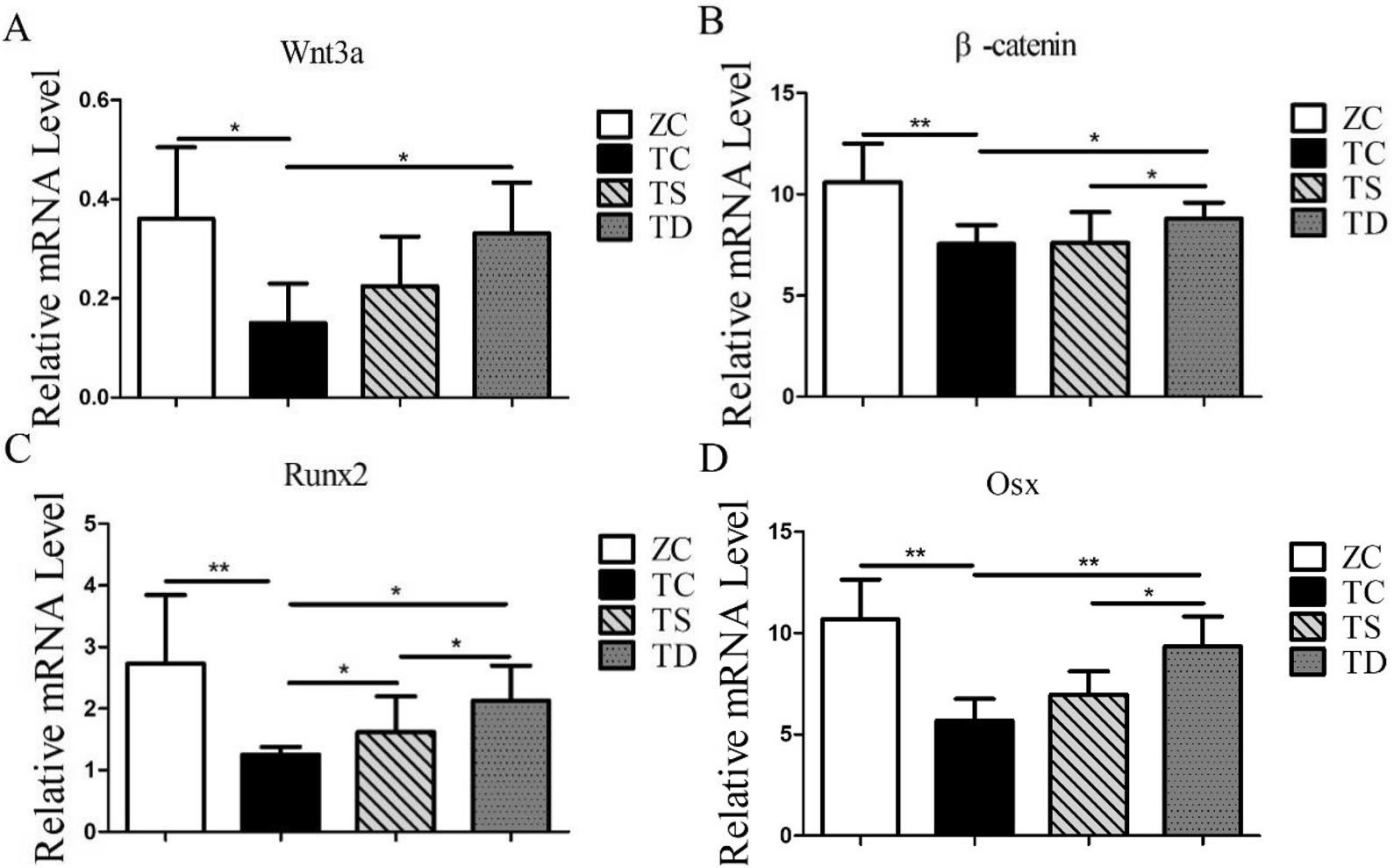
mRNA expression of related factors in Wnt/β-catenin signalling pathway. The data in the graphs are expressed as the mean  ±  SD, n  =  6. Relative mRNA expression levels of Wnt3a, β-catenin, Runx2 and Osx in the right tibia of each group of mice. *P  <  0.05, **P  <  0.01

**Fig. 6:**
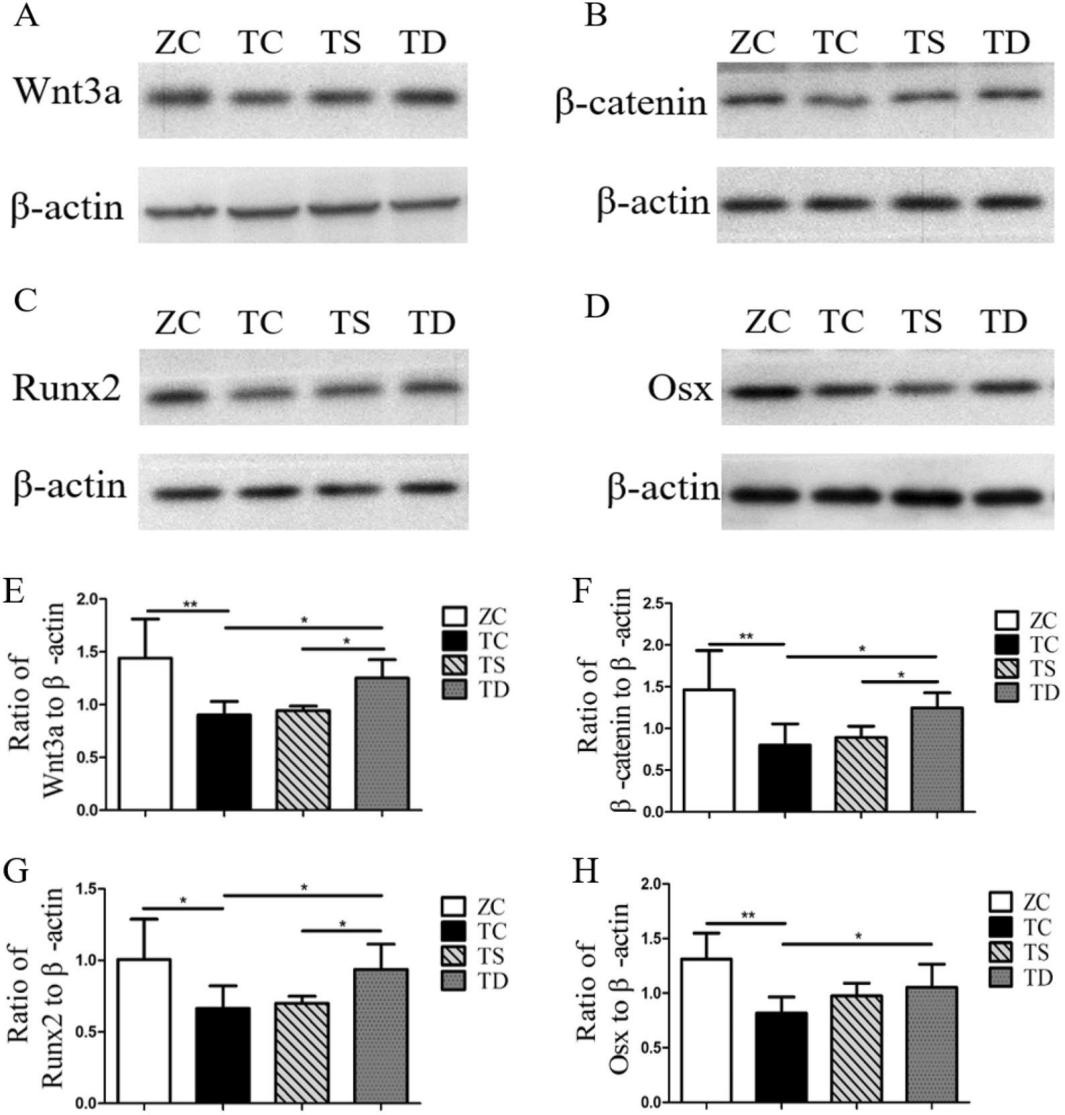
Protein expression levels of related factors in the Wnt/β-catenin signalling pathway. The data in the graphs are expressed as the mean  ±  SD, n  =  6. Femurs from six mice of each group mice were pooled for protein preparation. Western blotting was used to test the relative protein expression levels of Wnt3a, β-catenin, Runx2 and Osx. *P  <  0.05, **P  <  0.01

The expression levels of the related factors in the Wnt3a/β-catenin pathway, a key signalling pathway regulating bone formation, was tested, so as to investigate the mechanism of T2DM and exercise on bone formation capacity and bone histomorphology. T2DM inhibits OB differentiation and bone formation by inhibiting the Wnt3a/β-catenin pathway, leading to the degradation of the bone morphological structure and the initiation of osteoporosis. In the present study, the mRNA and protein expressions of Wnt3a (P < 0.05) (Fig. 5A), β-catenin (P < 0.05 or P < 0.01) (Fig. 5B), Runx2 (P < 0.05 or P < 0.01) (Fig. 5C) and Osx (P < 0.05 or P < 0.01) (Fig. 5D) were significantly downregulated. Thus, on the focus of the present study was on the effects of exercise on the expression of Wnt3a/β-catenin pathway factors in the bones of T2DM mice. In the TS group, the mRNA expression of Runx2 was significantly increased (P < 0.05) (Fig. 5C), but the mRNA expression levels of Wnt3a (P > 0.05) (Fig. 5A), β-catenin (P > 0.05) (Fig. 5B), and Osx (P > 0.05) (Fig. 5D), and the protein expression levels of Wnt3a (P > 0.05) (Fig. 5A), β-catenin (P > 0.05) (Fig. 5B), Runx2 (P > 0.05) (Fig. 5C), and Osx (P > 0.05) (Fig. 5D) had no significant changes. Compared with the TC group, the mRNA and protein expression levels of Wnt3a, β-catenin, Runx2, and Osx in the bones of the TD group mice were not significantly changed (P < 0.05 or P < 0.01) (Fig. 5). Additionally, aside from the Wnt3a mRNA and Osx protein, the expression levels of Wnt3a/β-catenin pathway related factors in the bones of the TD group were significantly higher than in the TS group (Fig. 6). Such results indicate that the Wnt3a/β-catenin pathway was suppressed in T2DM mice, and downhill running activated the Wnt3a/β-catenin pathway in the bone of T2DM mice.

## Discussion

T2DM is associated with skeletal system complications characterised by degenerated bone tissue morphology and osteoporosis [8, 45]. In the present study, using a high-fat diet in combination with STZ, a T2DM mouse model was established to investigate the effects of different exercises on bone formation. Inhibition of the Wnt3a/β-catenin pathway in the bones of T2DM mice was found to result in decreased OB differentiation and bone formation, leading to the degeneration of the bone morphological structure and decreased BMD. Such findings reveal that the Wnt3a/β-catenin pathway is a significant factor in T2DM inhibiting bone formation by decreasing OB differentiation and osteogenic capacity, and also reveal the success of the T2DM mice model in the present study. After 8 weeks of training, downhill running could improve the bone morphological structure and bone mass by promoting OB differentiation and the osteogenic capacity, which is regulated by activating the Wnt3a/β-catenin pathway, and the effect thereof on the bone formation of T2DM mice was better than swimming.

As a common complication in T2DM patients, osteoporosis is characterised by a degeneration of bone morphological structure and decreased BMD [26]. At present, exercise is a green and healthy intervention method that can promote bone formation and inhibit bone resorption, thereby improving bone metabolism [4, 12, 16]. Despite such benefits, existing studies have focused on the impact of exercise on the T2DM bone phenotype (such as BMD, biomechanical indicators, and others.). After 20 weeks of voluntary running, the maximum breaking force and stiffness of bone histology indexes in type 2 diabetic rats were improved [42], and 6 weeks treadmill exercise (10 m/min, 60 min/day, 5 days/week) could improve femoral BMD [1]. There is a scarcity of studies on the effects of different kinds of exercise on the morphological structure of bone tissue in T2DM mice. As such, swimming and downhill running were used to train T2DM mice in the present study. After 8 weeks training, compared with the TC group, the body weight was significantly decreased, but the other bone morphological structure indexes, such as tibia length, tibia wet weight, BMD, BV/TV, Tb.Th, Tb.N and Tb.Sp of cortical bone and trabecular bone in the TS group did not show significant changes. However, the body weight, tibia length, tibia wet weight, and morphological structure of trabecular bone in the distal femur of the TD group were all changed significantly.

Micro-CT was used in the present study to scan the distal femurs, and findings were made that the BMD, BV/TV, BS/BV, BS/TV, Tb.Th, and Tb.N of the trabecular bone in the TD group were all improved, but the cortical bone only exhibited changes in BV/TV, BS/BV and Tb.Th. Such findings suggest that downhill running could improve the morphological structure of bone tissue in T2DM mice, especially trabecular bone, while the effect of swimming was not significant. The positive effect of downhill running could be attributed to the higher-strength ground reaction (also known as direct mechanical stimulation) in the bones of T2DM mice during downhill running [38]. Direct mechanical stimulation promotes OB differentiation and bone formation, and inhibits OC differentiation, nucleation and bone resorption ability [40], and then improves the morphological structure of bone tissue, which is first manifested in the trabecular bone [32]. The effect of swimming on the morphological structure of the bone tissue in T2DM mice was found to be not significant, since there were no significant changes in the bone histomorphometry of the trabecular and cortical bones in the TS group, which could be ascribed to the mechanical stimulation on bone produced by swimming in the present study. Because findings were made in pre-experiments that the indirect mechanical stimulation of swimming on bone formation to improve the morphological structure of bone tissue was not significant, the mechanical stimulation of the bone of T2DM mice (i.e., muscle pulling) failed to reach the threshold of bone metabolism, leading to no significant improvements in the bone tissue morphology.

OBs are major cells that are differentiated from BMSCs and regulate bone formation metabolism [32]. Although T2DM inhibits OB differentiation and osteogenic capacity, exercise can promote the OB differentiation and osteogenic capacity of T2DM mice [30]. In the present study, after 8 weeks of exercise, ALP and Alizarin red staining were performed on OBs differentiated from BMSCs of the T2DM mice. The number of OBs and the osteogenic capacity in the TS group were found to have not significantly changed, while the TD group was found to have significantly changed. Moreover, the number of OBs and the osteogenic capacity in the TD group were significantly higher than those in the TS group. Such results reveal that downhill running significantly promoted OB differentiation and bone formation in T2DM mice, but the swimming effect was not significant. The positive effect of downhill running could be attributed to the direct mechanical stimulation of the bones produced by downhill running on T2DM mice, which can promote the phosphorylation of stress-activated protein kinases/Jun amino-terminal kinases (pSAPK-JNK) in bone [7]. After said signal pathway or cytokine expression is activated, the differentiation of BMSCs into OBs with strong osteogenic capacity is promoted [25]. The effect of swimming is not significant, and the muscle pulling force on the bone of T2DM mice is small, which cannot be related to the formation of effective mechanical stimulation of BMSCs, thereby inhibiting differentiation into OBs.

As a key signalling pathway regulating bone formation and metabolism, Wnt3a/β-catenin pathway has a significant regulatory function in OB differentiation and osteogenic capacity [36]. Ayse et al. [3] investigated the effects of interstitial fluid shear and tensile strain on bone cells and found that both mechanical stimuli promoted Wnt3a/β-catenin pathway activation. However, there are no existing studies on the promotion of the Wnt3a/β-catenin pathway by exercise and the regulation of OB differentiation and bone formation in T2DM mice. In the present study, RT-PCR and Western blotting were used to test the mRNA and protein expression levels of related genes in the Wnt3a/β-catenin pathway in bones of T2DM mice. The mRNA expression level of Runx2 was found to be significantly increased in the TS group, but the mRNA expression levels of Wnt3a, β-catenin, and Osx and the protein expression levels of Wnt3a, β-catenin, Runx2, and Osx had no significant change, while the mRNA and protein expression levels of Wnt3a, β-catenin, Runx2, and Osx in the bones of the TD group mice were significantly upregulated. Such findings indicate that downhill running can activate the Wnt3a/β-catenin pathway in the bones of T2DM mice, but swimming has no effect. The expression levels of related factors in the bones of the TD group were significantly higher than those in the TS group, aside from the mRNA expression levels of Wnt3a and Osx, which could be attributed to the role of Wnt and Osx at the translation level. The reasons for the differences in the aforementioned results are closely related to the large-scale direct mechanical stimulation of the bones produced by downhill running in T2DM mice. Large-scale direct mechanical stimulation can significantly upregulate BMP-2. When BMP-2 is activated, the interaction between Smad1 and DVL promotes β-catenin activity, thereby promoting Wnt3a-mediated OB differentiation and bone formation [27]. The mechanical stimulation of downhill running can also activate miR-495 expression in the bones of T2DM mice [41], and miR-495 activates downstream dishevelled 2 (DVL-2), while the expression of upregulated DVL will activate Wnt3a and downstream phosphorylation of β-catenin and Runx2 expression [9]. Limited by the small number of existing studies, the molecular mechanism of exercise affecting the Wnt3a/β-catenin pathway in T2DM bone remains unclear, and further study is needed.

## Conclusion

Bone formation is inhibited in T2DM mice, leading to osteoporosis. Downhill running activates the Wnt3a/β-catenin pathway in the bones of T2DM mice, promotes OB differentiation and osteogenic capacity, enhances bone formation metabolism, and improves the bone morphological structure.

## Data Availability

The raw data supporting the conclusions of this article will be made available by the authors, without undue reservation.

## Abbreviations

T2DM: Type 2 diabetes mellitus
BMD: Bone mineral density
OB: Osteoblasts
BMSCs: Bone marrow mesenchymal stem cells
HFD: High-fat-diet
OGTT: Oral glucose tolerance test
PFA: Paraformaldehyde
micro-CT: Microcomputed tomography
ROI: Region of interest
SD: Standard deviation
pSAPK-JNK: Phosphorylation of stress-activated protein kinases/Jun amino-terminal kinases
